# Impressions and Impression-Taking

**Published:** 1890-07

**Authors:** E. Bull


AMERICAN JOURNAL

—OF—

DENTAL SCIENCE.

VOL. XXIV. BALTIMORE, JULY, 1890. NO. 3.

ARTICLE I.

### IMPRESSIONS AND IMPRESSION-TAKING.

BY MR. E. BULL.

[Read before the Students' Society, London Dental Hospital.]

*Mr. President and Gentlemen*:—It is with great diffidence that I bring before your notice to-night the subject of impressions, recognizing, as I do, the supreme importance it bears to our profession as dental students. The value of an impression will be more fully brought home to us when we remember the fact, that no model can be an improvement on the impression; and too often the model and plate show the retrograde steps, which, should the impression remain untrue, will undoubtedly cause the denture to prove a failure.

I hope I may be able to introduce matter which some of you may consider controversial, and thus give rise to a good discussion, in which the many shortcomings of my paper may be, at least to some extent, compensated.

My intention is to deal, firstly, with the various materials used; secondly, to briefly describe the principal methods employed in the operation of impression-taking; and, thirdly, to discuss the question of selection of material.

Firstly, with regard to the materials.

Time was, when no impression materials were used, or impressions taken, but the unfortunate patient was made to sit in a chair for days together with a well-painted mouth, whilst a block of ivory was slowly let down on the spot. We opine a good fit resulted, since no better model could be had than the mouth itself, but we regard it as questionable whether the patient or the dentist had the worst of the bargain.

A Frenchman then discovered the excellence of wax as an impression material, and for many years this substance was exclusively used by dentists. From then up to the present time, various materials have been tried with varying success, but only four are now in common use, these being wax, modeling composition, gutta-percha, and plaster-of-Paris.

For a substance to be of any value in impression-taking, there are certain intrinsic and extrinsive properties that it must possess.

These are—

The necessary softness for taking the impression (*i. e.*, it must be soft enough to copy the finest lines of the mucous membrane, but must also have that consistency which will compress the soft tissues without displacing them.)

Must possess the proper hardness for retaining its shape when removed from the mouth.

Must take a sharp imprint.

Must remain where placed (*i. e.*, have no tendency to warp or fall away.)

Must harden promptly.

With undercuts must bend or break with moderate force.

Must be available under conditions existing in the oral cavity.

Must possess no qualities repulsive to patients.

The nearer a material attains perfection in these various properties, the better adapted it will be for our use.

WAX.—The wax used by dentists is common beeswax, which forms the framework of the comb. Virgin wax should be used, and its purity is an essential condition, care being taken that it is unadulterated with tallow.

There are two varieties of wax, yellow and white, of which the former is preferable, since the process of bleaching yellow to obtain white appears to destroy many of its good qualities.

Wax is prepared for the mouth by heating it in water hot enough to thoroughly soften without melting it. It may also be softened with dry heat, but this method is not so good as the former, since wax readily melts over the flame of a spirit lamp.

MODELING COMPOSITION.—This is the latest and in some respects one of the best materials for taking many sorts of impressions. It is composed of gum damar, stearine, French chalk, with carmine to color it, and a perfume to render it pleasant to the patient. The composition varies in its consistence according to the amount of stearine and chalk introduced into it.

Modeling compound is best softened by dry heat over a spirit lamp, as water appears to injure its consistency. The impression tray should also be heated, and the composition, rolled into a ball, applied to the palatal portion, and kneaded from thence to the rim. By this means a good surface free from creases is obtained, and this may be again surface-heated over the lamp before application to the mouth.

GUTTA-PERCHA.—Gutta-percha is prepared from the juice of the *isonandra gutta* tree. It should be used in a pure state, as foreign substances tend to diminish its plasticity. Water heated to about 180° Fahrenheit should be used to soften gutta-percha. It must be worked with moist fingers, and before introduction into the mouth, its surface should be chilled in cold water; this is important, since should this precaution not be observed, the patient will be put to some considerable amount of pain owing to the contact of the hot material with the mucous membrane.

PLASTER-OF-PARIS.—Plaster-of-Paris is manufactured from gypsum, which is ground and calcined to drive off water. It should be perfectly dry, and there is one essential condition that must be complied with in order to obtain the best results—it must be properly mixed.

The setting of plaster is a chemical process, two molecules of water being taken up to one of plaster. The nearer this proportion is arrived at, the more satisfactory will be the result. It should be mixed in a small bowl, the plaster being sprinkled in until it is entirely taken up by the water, and the mixture is of a medium thickness; it should then be used immediately.

Since pure plaster requires some time to set, it is found advantageous to introduce something to hasten the process. Of the many agents used, potash-alum, ten grains to a tablespoonful of plaster, is by far the best. Common salt is also strongly recommended, and its presence is by no means unpalatable to the patient.

It is also very useful to mix some coloring matter, such as rose pink, with the modeling plaster, so that we may the more readily distinguish our plaster impression from the true model when chipping it off during the operation of casting.

We must give a passing word to the various sorts of impression trays that are in general use. Years ago, trays were commonly made of silver, but now-a-days they get so battered about to meet the requirements of each particular case, that it is unadvisable to employ any valuable metal in their manufacture. Britannia metal (tin alloyed with lead and bismuth) makes good trays, and is probably as useful as any other material. A complete set should be kept, the operator selecting those that best suit his particular method of treatment.

Practitioners of fifty years ago found fifteen trays amply sufficient, and Mr. Harris speaks with apparent complacence of having in one instance been absolutely obliged to make a special tray in order to obtain a correct impression. But the exigencies of prosthetic dentistry, as practiced to-day, have altered this pleasant state of affairs. It is now necessary in most cases where we are using plaster to take the impression, and in many a case beside, to make a special tray. I have no time now to go into the details of their manufacture, but I may say they may be made by such methods as striking up a piece of Britannia metal, or pouring tin.

So much for the materials employed. We will now go on, *secondly*, to the operation of impression-taking. We will first confine ourselves to general methods, and then particularize a few of the difficulties we meet with, and the means by which such difficulties may most readily be overcome.

The preparation of the mouth scarcely comes under the province of this paper, but I may mention, *en passant*, that all irritants, such us salivary calculus, roots and diseased teeth that will not yield to treatment, must be removed, and the gums and mucous membrane brought into a healthy condition, before a denture should be made, or an impression taken.

METHOD OF USING WAX, COMPOSITION, OR GUTTA-PERCHA.—We may take these three materials together, as there is little essential difference in the *modus operandi*.

Before taking the impression, the mouth should be dried with a soft napkin, and should the secretions be copious or the gum spongy, it is advisable in some cases to make use of an astringent, such as dilute phenol sodique, for some days prior to the operation.

The position of the operator should be behind and to the right of the chair, and he should be so placed that he can command a full view of the interior of the patient's mouth. The tray and material may best be introduced into the mouth by distending the left side of the lips with a finger of the left hand, pressing the tray against the right side, and passing it in with a rotative movement. The cup should then be carefully adjusted over the arch without disturbing the material, and then pressed up until all the parts are imbedded. The patient may then be instructed to draw down the upper lip, and external pressure on the projecting material must be applied all round the alveolar ridge with the finger, an operation especially important with undercuts. With a good impression, the atmospheric pressure will be great, and difficulty will be experienced in removing the tray, this being best effected by elevating and depressing it with quick firm movements, thus causing the introduction of air between the palate and impression.

The material should be fairly hard before removal is attempted, and the setting may be hastened by the application of ice-cold water on a napkin. Owing to the smallness of the mouth, or the strength of the orbicularis oris, it will often be found difficult to introduce the tray. This difficulty must be overcome with gentleness, the use of force only tending to make matters worse. With irritability of the fauces and adjacent parts, causing retching and uneasiness, we should gently paint these regions with a camel's hair brush, with camphor water, or employ a gargle of the same.

METHODS OF USING PLASTER.—In the use of plaster, the patient should be placed upright in the chair, with his head inclined slightly forwards; the breathing may safely be left to itself, but he should be directed not to swallow during the operation. The drying of the mucous membrane is especially important before taking a plaster impression. The cup having been introduced into the mouth, should be pressed up with the rear slightly in advance of the front, in order to prevent any plaster escaping at the heel and causing irritation of the fauces, or dropping down the throat.

It is especially important that the back edge of the tray should fit the palate when plaster is used for the impression.

If it does not, a bridge of soft wax should be built across it, conforming in shape to the palate.

It usually takes from three to four minutes for the plaster to set, and no attempt at removal should be made until a clean fracture results on breaking a portion of the surplus in the bowl. The tray may then be withdrawn as in wax impressions. Fracture of the plaster will usually result, and the pieces must be carefully collected and fitted on to the tray.

Instead of making a special tray for the use of plaster, another method may be adopted. A rough impression in wax is taken, and a layer varying in thickness from ¼ to ⅛ of an inch, tapering towards the rear to prevent a surplus of plaster in that region, is trimmed from this. The wax should then be deeply scored and undercut, a thin surface of plaster run on, and another impression taken.

We will now describe a few of the more difficult cases to be met with in impression taking.

The most common of these is a dovetail space between two teeth. Two or three excellent methods of operating are open to us.

The offending teeth may be dried, and a small piece of wax fitted on to the undercut portion; this must be well vaselined before taking the impression, or if plaster is being used, wax cut-offs may be employed, causing a fracture of the plaster down the middle of the dovetail.

If the case be such that a complicated fracture is unavoidable, a good plan is to fill such spaces with plaster, which when set must be trimmed so that no undercut exists and it should then be coated with gum sandarac. Having taken the impression, the portion in the dovetail may easily be removed by slitting it transversely almost to the gum, when it may be fractured without being defaced, and fitted into the impression.

Another common difficulty is a deep undercut. Wax and composition are of little use, although gutta-percha may meet the requirements of the case, but undoubtedly, particularly if the mouth is edentulous, plaster is the material to use, and it is in these cases that wax cut-offs are extremely serviceable. Strips of wax are placed in the tray, forming a ridge corresponding to the alveolar process of the jaw; two transverse strips may also be placed at points corresponding to the position of the canines. The cup, being oiled, parts from the plaster, is allowed to remain in the mouth. This, when set, is broken off by the operator, the cut-offs anticipating the fractures, and allowing him to remove the whole impression in four or five large portions. The palatal portion may be removed with a special hooked instrument.

There is an excellent method of procedure in taking plaster impressions where only one or two teeth are standing, in which the use of cut-offs is avoided. Strike up the tray so that it touches the crowns of the standing teeth, proceed as above, and the pieces of plaster will fracture across the weak spots above the teeth.

A difficulty often presents itself in mouths where there is protrusion of the front teeth. An ingenious tray has been invented by Mr. David Hepburn, to meet these cases, called after him, "Hepburn's Sliding Section Tray." It consists of an ordinary tray, with the rim in front cut away, this portion being made to slide along the handle, so that when pushed up it comes into its normal position. The method of operating is to take an impression in composition with the body of the tray, not allowing the material to overlap the protruding teeth; a piece of composition is then placed on the sliding rim, which is pushed home. The two portions may be readily removed, separately, with dragging.

I may also briefly mention here, Kingsley's method of taking impressions for artificial palates.

When the palate is merely perforated from accidental causes, no special apparatus is required, as only the boundaries of the foramen need to be defined. Great care must be taken, however, in these cases, not to use too much impression material, since the surplus may be pushed up into the nasal cavity, in which case great difficulty will be experienced in removing the tray.

But with congenital clefts, it is essential that the entire borders of the fissure from the apex to the uvula, also the form of the cavity above the palate should be perfectly represented in the model.

An impression of the lingual surface must first be taken, plaster being used with the greatest success. The next step is to take an impression of the nasal surface of the hard palate. This can be done by filling the lower portion of the cavity above the roof of the mouth with soft plaster, and while it is yet quite soft, carrying the palatal impression against it, having first soaped its surface to prevent adhesion between the two. The two portions may be easily removed separately, the nasal part being carried backward and withdrawn from the mouth with a suitable pair of forceps. The irregular surface of contact indicates their relations when the two are brought together.

Let us, *thirdly*, discuss the relative merits of the various materials used in impressions.

It appears to me in impression-taking, as in all other branches of our profession, we are too apt to settle down into a routine, and from either laziness or want of enterprise, we jog along in a circumscribed area, enunciating and practicing our own views, and thus losing sight of or entirely disregarding the many advantages which may attend another style of operation.

Gentlemen, I can only bid you beware of conservative methods; it behooves us to move with the times, and to make ourselves masters of new theories and new practices, although undoubtedly we should assure ourselves of their advisability and efficacy before bringing them into general use.

I do not hold with the modern craze for plaster impressions. I am quite aware that we shall be told in the ensuing discussion that men taking wax and composition impressions cannot even compete with those who use plaster. Well, all I can say is, that wax and composition have competed and do compete at the present day with plaster, and what is more, with the greatest success. Do not misunderstand me; I would not eschew the use of plaster for impressions. On the other hand, I think nothing else can be used in some mouths, such as edentulous jaws with deep undercuts, and cleft palate cases.

It speaks well for wax, that for so many years it was the only impression material used, and although times have changed, yet the callow student of to-day, who uses plaster indiscriminately for everything, would be perhaps surprised at the sharp impression that can be got by a practitioner skilled in the use of wax and composition.

It is the abuse, rather than the use, of wax that has brought it into apparent disrepute with some.

Gutta-percha is extremely serviceable, but it appears to want a great deal of practice for anyone to become proficient in its use. The great virtue of this material lies in its elasticity and power of regaining its original shape, which allow of its employment in undercuts, where wax or composition would be practically useless. Gutta-percha shrinks very considerably in cooling, and hence is used by some in taking impressions for regulation plates, thus securing a tight fit.

In selecting our material, there are two conditions to be borne in mind; a minimum of inconvenience to our patient, and a maximum of convenience to ourselves. For whatever we do, we must remember that a patient seldom feels at home in the operating chair, and anything which will tend to increase his discomfort must be avoided, as far as is compatible with good practice. In this fact lies one of my great objections to plaster as an impression material, for let men say what they will, plaster is very obnoxious to the patient, even with the greatest care. I advisedly leave incompetency out of the question, but probably in nothing is bad operating more painfully apparent, or more discomforting to the patient, than in taking a plaster impression.

Again, our own convenience is to be considered. With the plastic materials we have very little trouble, but with the plaster impression, the necessary preparation, together with the striking up of a special tray, and the subsequent fitting together of the fragments, are sources of great loss of time, and I fear too often of tempter as well.

To sum up these remarks, as a general rule I recommend the use of wax, composition, or gutta-percha, since they are the most comfortable to the patient, and the most expeditious for ourselves, resorting only to plaster when these materials can not be used with any hope of success.

In conclusion, I have to thank you for your kind attention, in listening to my attempt to discuss a subject which after all is more practical than theoretical.—*The British Journal of Dental Science.*

---

ARTICLE II.

